# Lipid-lowering effect of maize-based traditional Mexican food on a metabolic syndrome model in rats

**DOI:** 10.1186/1476-511X-12-35

**Published:** 2013-03-15

**Authors:** Juan Manuel Muñoz Cano, Andrea Carrillo Aguilar, Juan Córdova Hernández

**Affiliations:** 1 Universidad Juárez Autónoma de Tabasco, División Académica de Ciencias de la Salud, Avenida Méndez 2838-A, Villahermosa, Tabasco, CP 86150, México

**Keywords:** Whole grain cereal, Blood lipids, Blood glucose, Alanine amino transferase

## Abstract

**Background:**

Maize-based food is typical in Mexico and other Mesoamerican countries. Used for millennia, they have recently been replaced by modern food that is associated with an increase in the prevalence of non-communicable chronic diseases. This study was carried out in order to evaluate the effects of traditional food on lipid profiles.

**Methods:**

Metabolic syndrome was induced in animals given a 30% sucrose solution. The animals were given maize tortillas (n=5) and maize pozol (n=5), traditional Mexican food items. A control group was given a 30% sucrose solution in the laboratory diet (n=5) and a witness group was given plain water and pellets. Triglycerides, cholesterol and glucose in tail blood were recorded each month between weeks 12 to 24. Blood was obtained from the cardiac cavity on week 28 and triglycerides, total cholesterol, LDL, HDL, C-reactive protein, alanine amino transferase, glucose and glycated hemoglobin were recorded.

**Results:**

The animals provided with supplementary traditional food presented a lower increase in triglycerides up to week 24 (p<0.001). Data recorded on week 28 showed lower values of LDL (p<0.05), a lower percentage of glycated hemoglobin when maize tortillas were provided (p<0.01) and lower values of alanine amino transferase when both food items were provided (p<0.01).

**Conclusions:**

Providing traditional Mexican food generated a protective effect against the intake of a 30% sucrose solution over a long period.

## Background

The present pandemic of overweight and obese people is related to the increase in the prevalence of non-communicable diseases (NCD), specifically of diet-related chronic diseases that have caused 65% of global mortality [1]. Although being overweight is related to an increase in the amount of food that is ingested, independently of what is eaten, an obesogenic environment is favoured by the availability of very processed, high palatability and energy dense food and drinks. In contrast, populations that maintain their feeding patterns, mainly based on wild food items [2] and on the traditional preparation of the characteristic cuisine of a community, like Mediterranean [3-5] and Japanese food [6,7], present less problems related to being overweight, including NCD. Several studies have recorded the beneficial effects of eating food based on whole grain cereals, and its relationship with a lower risk of acquiring NCD [8,9].

Recent findings on food and metabolic profiles have shown that, apart from the macro and micronutrients (proteins, carbohydrates, lipids, vitamins and minerals), wild and little processed food has components that are not nutritious, as they do not provide calories, but that modulate the metabolic profile or act protectively against diseases, particularly the NCD. These elements, known as bioactive components, inhibit or activate genes that modify the metabolic response to macronutrients and increase the feeling of satiety. Mexican epidemiological studies have shown than people with eating patterns that include traditional food items, under similar conditions of body volume, have a lower risk of developing type 2 diabetes mellitus [10-12]. The basis of Mexican traditional food is maize prepared by the process of “nixtamalización” which conserves the properties of the whole grain cereal [8]. Maize has been found to decrease the risk of developing NCD [8,13-15]. In view of this, the purpose of this study was to examine the effect of traditional Mexican food on an animal metabolic syndrome model.

## Methods

### Animals

The protocol complied with an adequate handling of the experimental animals as is described in the NOM-062-200-1999 that states the technical details for the production, care and use of laboratory animals for the year 1999. The female Wistar rats with 4 weeks of age presented initial weights of 90–100 g. The brand of boxes used for this experiment where EHRET® (Germany), which are made of polycarbonate, with 33 cm wide and 55 cm long and with a depth of 20 cm, with grate grids of stainless steel. The drinking troughs were EHRE™ (Germany) of 500 ml, made in polycarbonate with stainless steel tips (for autoclave sterilization). Five animals where introduced in each box with and ad libitum access to food and water. The food of the experimental animals was 2018S Teklad Global 18% Protein Rodent Diet® (Harlam Laboratories®, USA). This food has an energy density of 3.1 kcal/g of which 18% comes from lipids. The flour to prepare the maize-based foods was manufactured by Maseca® (Gruma®, Mexico). The flour energy density is 4.5 kcal/g of which 12% comes from lipids. The animals were subjected to cycles of 12 hours of light and 12 hours of darkness, temperature of 22-24°C and humidity of 60 to 80%. The rats were kept in the Unidad de Producción, Cuidado y Experimentación Animal (UPCEA) of the Division Académica de Ciencias de la Salud, Universidad Juarez Autonoma de Tabasco. The study was approved by the Research Committee and the Bioethics Committee of the institution.

### Supplementation

Metabolic syndrome was induced in three groups of Wistar rats by giving them water with 30% sucrose (Sigma-Aldrich, St. Louis, Missouri, USA) to drink, following the method of El Hafidi [16,17]. The experiment started when the rats were four weeks of age and ended 28 weeks later. The experimental animals were distributed in three groups. The first group received maize tortillas and pellets (n=5), the second received maize pozol [18] and pellets (n=5), the third was the control group (n=5) and received a 30% sucrose solution and no supplemented traditional food in their lab diet. There was also a witness group that received plain water and pellets (n=5).

Following processes inherent to the retrogradation of the maize-based food, it was decided to provide it at precise periods of time, after which the animals returned to their lab diets. An important point is that pozol favours bacterial growth, mainly of lactobacilii, that seem to present hypotriglyceridemic activity [19]. For supplementation, the animals of the tortilla and pozol groups were distributed in individual boxes with no hay and were allowed to eat traditional food for four hours of each day at a ratio of 50 g of each type for each animal, according to the group. After the defined period of time, the food remains were removed to quantify intake. The animals were then re-grouped in the boxes and provided with standard experimental rat food at libitum for the rest of the day in order to minimise the risk of deficiencies in macro or micronutrients, especially of the amino acids lysine and tryptophan.

The tortillas and pozol were prepared with Maseca® (Gruma®, Mexico) maize flour following the manufacturer’s instructions. The product is made from cooked corn kernels with lime (nixtamalization), dried and ground for sale as flour. For both foods the base is a mixture of 500 ml of maize flour with 300 ml of water. The mixture was made in an Oster® stand mixer with 350 watts motor, with a 4.5 qt gallon capacity, and a bowl of 22 × 35 × 35 cm. Once the mixture is homogenized, portions of 30 g were made, which were placed between cellophane sheets and were flattened with a rolling pin with which the tortillas were formed. The cooking was done in a Rodotec® NG50, commercial tortilladora. The cooked procedure is entering to the dry-heat oven on a conveyor belt with comales, which are on rollers, so the tortillas are recovered in the exit door.

Pozol is a “nixtamal” dough prepared with “nixtamalised” maize in traditional establishments. This maize dough is cooked and ground with lime and water, to which is added toasted and ground cocoa, obtaining a dark dough that is diluted in water. The dough for the experiment was prepared with Maseca® (Gruma®, Mexico) maize flour, as were the tortillas, mixing 125 g of maize dough and 9.6 g of toasted and ground cocoa. The corn dough and the cocoa grounded were mixed in an Oster® stand mixer.

### Blood collection and biochemical variables

Data on the intake of 30% sucrose and traditional food were recorded daily. The weight of each animal was recorded before breakfast every week. Between weeks 12 to 24 blood was obtained from the animals’ tails before breakfast for a monthly recording of glucose, cholesterol and triglycerides using test strips in an Accutrend® Plus/GCT system (Roche Diagnostic GmbH,® Mannheim, Germany).

At week 28, the animals were anesthesised and 1 ml of blood was obtained from the cardiac cavity using BD Microtainer® (Becton Dickinson, Franklin Lakes, NJ) non-additive tubes, in order to measure glucose, triacylglycerols (TAG), total cholesterol (TC), low density lipoproteins (LDL), high density lipoproteins (HDL), C-reactive protein (CRP) and alanine amino transferase (ALT). A second 1 ml of blood was obtained and placed in Microtainer® (Becton Dickinson, Franklin Lakes, NJ) tubes with K_2_EDTA BD to record glycated hemoglobin (HbA1c) and carry out a hematic biometry. The animals were then placed in a CO_2_ chamber to die.

An automated Johnson & Johnson Ortho-Clinical Diagnostics® (Rochester, NY, USA) equipment with a clinical chemistry system was used to measure glucose, TAG, TC, LDL, HDL, CRP and ALT. Glucose was evaluated following the criteria of the American Diabetes Association [20], where ≤5.54 mM/L is desirable, 5.6-6.93 mM/L indicates pre-diabetes and ≥6.94 mM/L indicates diabetes. Lipids were evaluated following the criteria of the National Cholesterol Education Program [21]. TAG were considered desirable with ≤1.68 mM/L, borderline high with 1.69-2.25 mM/L and high with ≥2.26 mM/L. TC was considered desirable with ≤4.39 mM/L, borderline high with 4.4-5.16 mM/L and high with ≥5.17 mM/L. LDL were optimal with ≤2.83 mM/L, near optimal with 2.84-3.35 mM/L and borderline high with ≥3.36 mM/L. ALT was defined as desirable up to 0.6668 Kat μ/L. HbA1c was measured using a Stanbio Lab® (Boerne, Texas, USA) work kit and a JENWAY 6305® (Staffordshire, UK) spectrophotometer. The absorbance ranges were calculated following the manufacturer’s guidelines for each standard and sample. The reagents for the determinations were from Ortho Chemical Diagnostic® (Rochester, NY, USA).

### Statistical analyses

A two way ANOVA was applied following a Dunnet procedure to determine significance for the values of the tests. The SigmaStat programme (Systat Software®, USA) was used to prepare the figures. The statistical significance was established at a P<0.01. The results are presented with means and standard deviation (SD).

## Results

The animals in the experimental groups recorded no difference in the weight means after 28 weeks, nor did they present differences in the intake of the sucrose solution (data not shown). The glucose and cholesterol values tended to increase, with no differences among groups (data not shown). Data on tortilla and pozol intake are presented in Table 1.

**Table 1:** Food supplement intake

**Week**	**8**	**12**	**16**	**20**	**24**
Tortilla M(SD)	16.93(4.77)	14.59(4.89)	10.52(5.47)	12.57(4.88)	10.37(5.97)
Pozol M(SD)	12.16(5.81)	13.58(6.02)	12.4(5.1)	12.7(5.12)	15.91(6.97)

The animal consumption of maize pozol and maize tortilla for four hours every day is presented. The result of substracting the left-over food from the food provided daily. The average in grams of one day’s intake in the corresponding week is shown. M = medium, SD = standard deviation. The rest of the day they were fed ad libitum with 2018S Teklad Global 18% (Harlam Laboratories®, USA). The animals of the “control” groups and “metabolic syndrome” daily received the food ad libitum.

**Figure 1:**
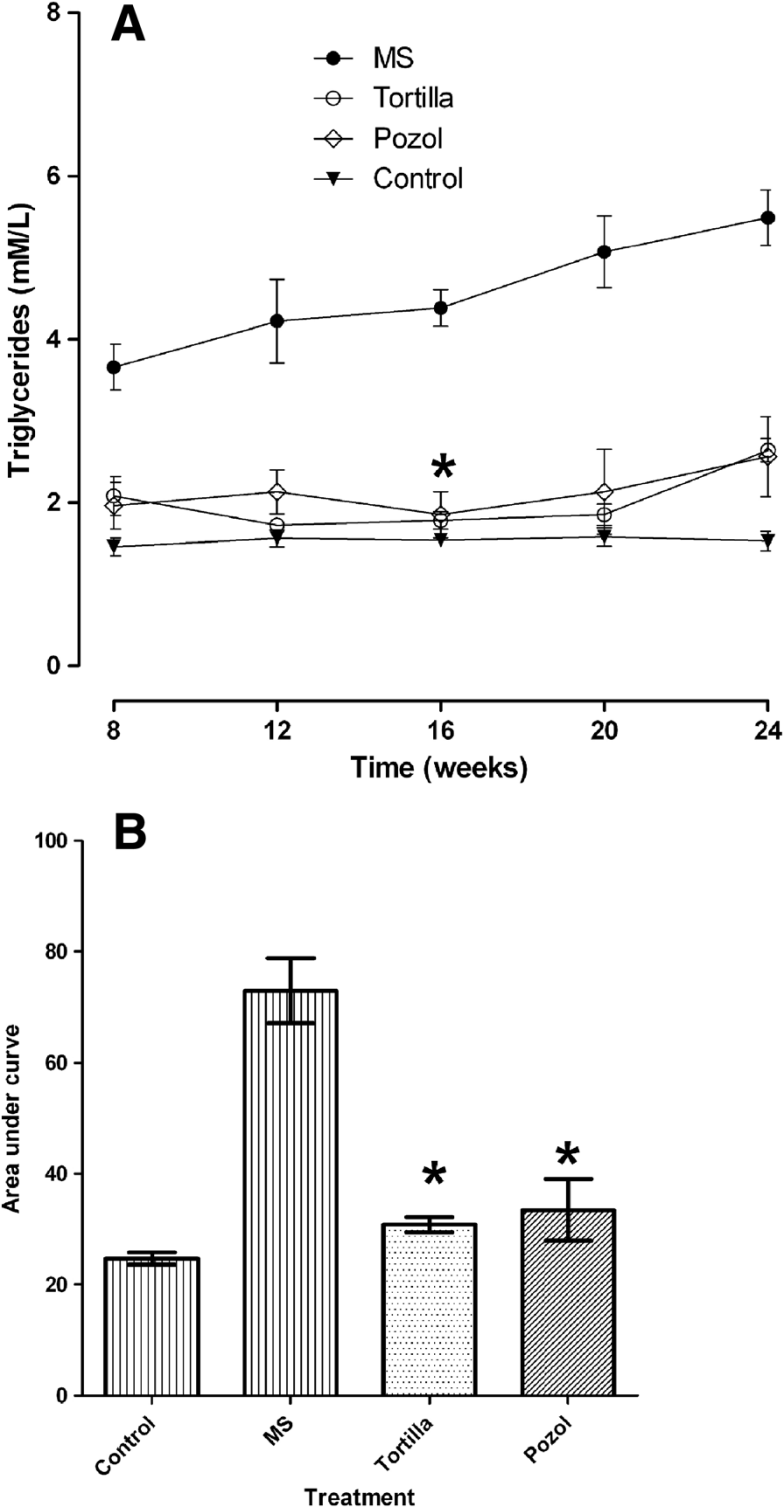
**Effect of traditional food on plasma triglycerides (mM/L).** Panel **A**, each point is a mean ± SD for n=5, for week 8 to week 24. Panel **B**, area under the curve for weeks 8 to 24. MS, metabolic syndrome=30% sucrose, Tortilla=30% sucrose and maize tortilla, Pozol=30% sucrose and maize pozol. The average of the values obtained from blood of the animals’ tails. *Significant difference for with and without traditional food, P<0.001. No significant difference for control and with traditional food, P<0.05.

Triglyceride values also tended to increase; however, the difference between the curves for the time from week 8 to week 24 from the blood samples taken from the animals’ tails, the rats given a sucrose solution (metabolic syndrome, MS) and the rats provided traditional food, was highly significant (P=0.001). This was more evident when analysing the area under the curve for these values where the means, and the error bars on each column show the difference more clearly (Figure 1). The average of the values from week 8 to week 24 of glucose and total cholesterol did not show big differences between groups (data not shown).

**Figure 2:**
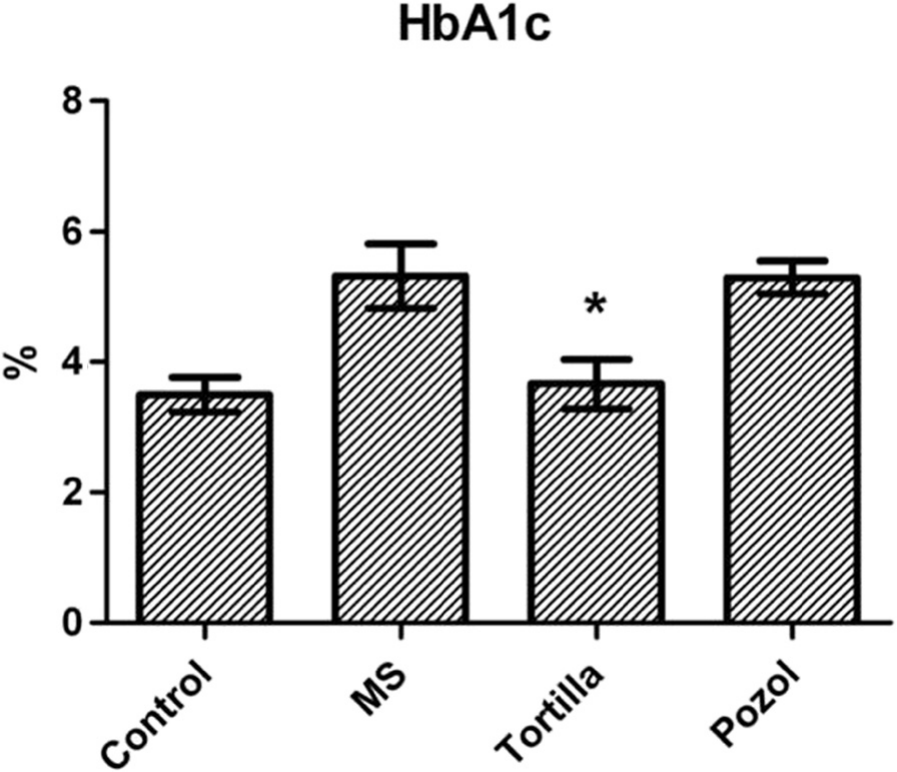
**Effect of traditional food on HbA1c.** HbA1c=glycated hemoglobin. MS, metabolic syndrome=30% sucrose, Tortilla=30% sucrose and maize tortilla, Pozol=30% sucrose and maize pozol. *Significant difference for with and without maize tortilla, P<0.01.

The addition of traditional food did not affect the average values of glucose in plasma on week 28 when they were compared with the values of the metabolic syndrome group. However, the HbA1c presented a significant difference between the group that received maize tortilla, with a mean of 3.7% ± 0.4, and the other two experimental groups, with 5.3% ± 0.6 for the group with no supplements (MS) and 5.3% ± 0.3 for the group that received maize pozol. The difference was significant (P=0.01) and the value was similar to that of the control group for which a mean of 3.5% ± 0.3 was recorded (Figure 2). No significant differences were recorded among the groups for the values of mean globular volume, mean globular hemoglobin and mean globular hemoglobin concentration (data not shown).

**Figure 3:**
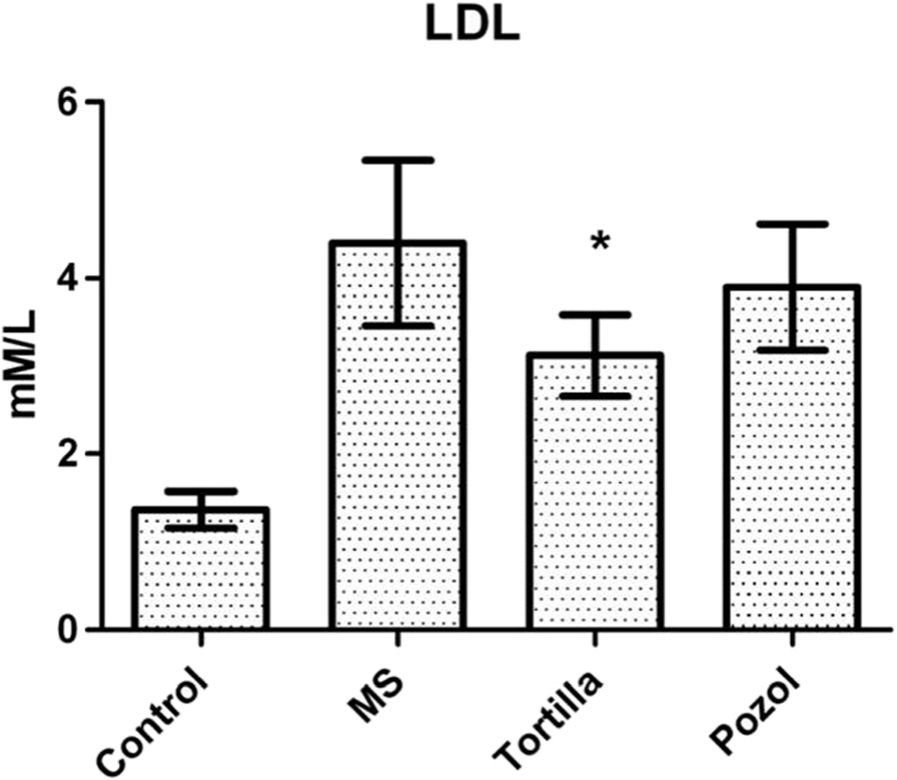
**Effect of traditional food on LDL.** LDL=low density lipoprotein. MS, metabolic syndrome=30% sucrose, Tortilla=30% sucrose and maize tortilla, Pozol=30% sucrose and maize pozol. *No significant difference for with and without maize tortilla, P<0.05.

Cholesterol increased in all the experimental groups on week 28 (S30%=7.1 mM/L ± 0.8, S30%+To=5.7 mM/L ± 1.0, S30%+P=5.8 mM/L ± 1.7) in comparison with the control group (3.5 mM/L ± 0.5). No significant difference was obtained when the cholesterol values of the groups provided traditional food were compared with those of the groups without this food (data not shown). A significant difference (P<0.05) was recorded for the LDL values, where the group with no supplements (MS) had an average of 4.4 mM/L ± 0.95, the group provided with maize tortilla had 2.99 mM/L ± 0.54 and the group provided with maize pozol had 3.9 mM/L ± 0.85 (Figure 3).

**Figure 4:**
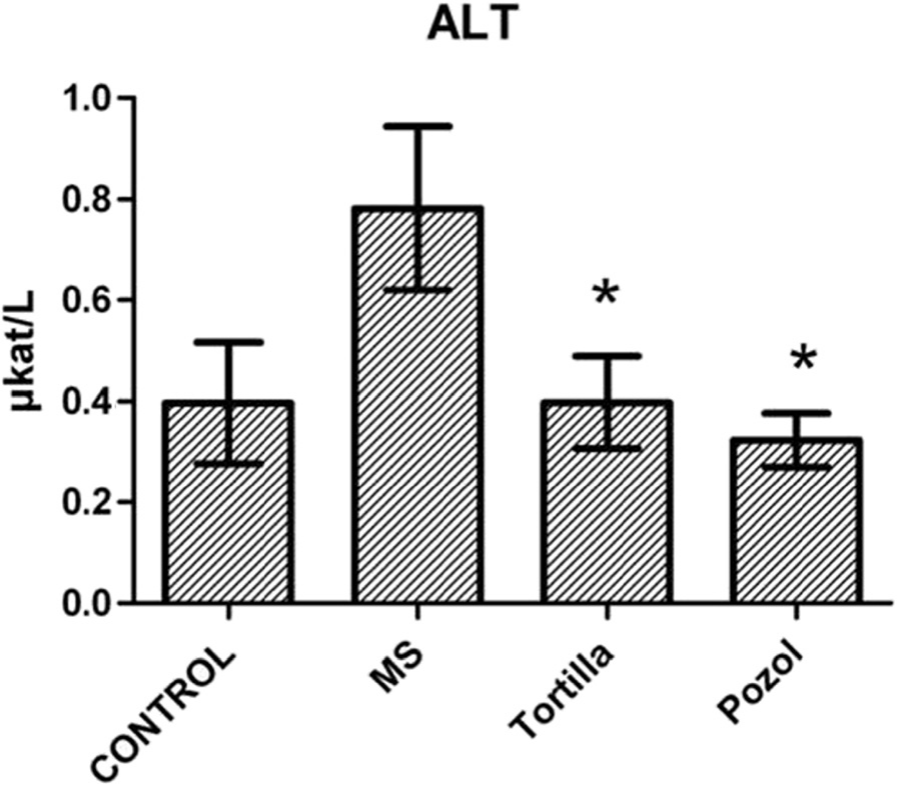
**Effect of traditional food on ALT.** ALT=alanine amino transferase. MS, metabolic syndrome=30% sucrose, Tortilla=30% sucrose and maize tortilla, Pozol=30% sucrose and maize pozol. *Significant difference for with and without traditional food, P<0.01.

No significant differences were recorded for the CRP values (data not shown). The analysis of the ALT values indicated an increase in the group provided with sucrose and no traditional food (MS), with an average of 0.78 μkat/L ± 0.16. This value, although below the cutting limit, is significantly different (P<0.01) from those recorded for the groups that received maize tortilla (0.40 μkat/L ± 0.09) and maize pozol (0.33 μkat/L ± 0.05), which in turn are similar to the control group (0.40 μkat/L ± 0.12) (Figure 4).

## Discussion

The results obtained showed that both maize tortilla and maize pozol lowered triglycerides and LDL, in spite of a high intake of sucrose solution (Figures 1 and 3). Persistent insult moved towards metabolic syndrome, however, the animals that fed on these traditional foods had less damage, as shown by the biochemical variables among which the values of the lipids presented significant differences in the last week of the experiment.

Maize tortillas and maize pozol had different effects on the modulation of glucose levels in plasma. Maize tortillas are prepared with maize dough, the “masa”, on a traditional hot metal surface, the “comal”, or in industrial “tortilladoras”, whereas maize pozol is simply dissolved and drunk. The different preparation processes may modify the composition of the components [22], including the concentration of resistant starch [23] or of phenolic compounds [24]. Notwithstanding that the values of glucose in plasma showed no differences on week 28, differences were recorded for HbA1c (Figure 2). It must be remembered that while the evaluation of glucose in plasma is transversal, that of HbA1c is more dynamic as it records a glucose average during the half life of the red blood cells.

The protective effect of maize starches on the liver has been demonstrated in models of liver ischemia-reperfusion [25]. A high intake of fructose, present in sucrose, is associated with an increase in global mortality caused by non-alcoholic liver damage. Both maize tortillas and maize pozol presented a protective effect against the hepatotoxic effect of the sucrose solution, as the means of the ALT values in these groups were similar to those in the witness group, whereas the means of this enzyme in the plasma of the group with no supplementary traditional food increased (Figure 4).

Considering that this study applied the culinary preparations that people have used for millennia, it contributes to recommending the food that may be beneficial to most of the population within a geographical region. The retrieval of traditional cuisine dishes is an attempt to apply the findings of epidemiological studies and connect them to sustainability [26] and sociocultural-ecological [27] approaches, in order to design better recommendations based on specific food items [28] and diminish the high or increasing burden of disease or death associated with unhealthy diets, for the prevention and control of NCD [29].

The results of this experiment also show that, in a model more similar to the conditions in which NCD develop, the intake of specific food items, though decreasing the effects of the environment in the form of a sucrose solution, in the long run produces the metabolic changes of the metabolic syndrome, even in the presence of protective foods. The value of these results must be placed in the context of recommending types of food as part of a way of life, more than in recommending a particular food item to treat hyperlipidemia or to decrease the risk of developing type 2 diabetes mellitus or hypertension [30,31].

The contribution of this study lies in the need to elaborate recommendations for a healthy nutrition based on meals, not individual nutrient items [28]. This emphasis considers that they are not equivalent [2] and that all culinary preparations made with one individual food item do not have the same effect on metabolism and health [31]. Experimental studies with humans have shown that the way one cooks is important, as in the case of black beans with respect to glucose metabolism [32]. The way fish is prepared also has an effect on the prevention of cardiometabolic, cerebral vascular and Alzheimer’s diseases, according to epidemiological studies [33,34]. Thus, it is important to analyse the effect of maize tortillas on achieving a healthy phenotype, not only for Mesoamerica where there is a high intake of this food, but to establish a line of research to analyse the effect of food preparation in traditional kitchens on the metabolic profile of the population.

This study on tortillas and pozol, traditional Mexican food items, does not define whether the changes are the result of the metabolism of probiotics, of the metabolic activity of prebiotics [19,35] or of changes in chromatin methylation, among other possible mechanisms. However, it is a first study aimed at establishing the molecular modifications that govern the genes that are expressed and those that remain silent, and determining the reason behind the presence of healthy obese phenotypes and of obese phenotypes associated with diseases following food intake patterns.

## Conclusion

Supplementation with maize tortillas and maize pozol protected against the high intake of sucrose in a long term metabolic syndrome model.

## Competing interests

There are no competing interests regarding this manuscript.

## Authors’ contributions

JMMC designed the experiment and is primarily responsible for the preparation of the manuscript. ACA carried out the laboratory analyses. JAC analysed the data. All authors contributed and approved the final manuscript.

## Authors’ information

JMMC is a researcher at the School of Medicine of the University of Tabasco, Mexico. He graduated in Molecular Biomedicine. He attended pregraduate courses in medical education. ACA is a Master’s in Science student at the University of Tabasco, Mexico. JAC is a Medicine professor at the School of Medicine of the University of Tabasco, Mexico.
